# Proteins and Amino Acids from Edible Insects for the Human Diet—A Narrative Review Considering Environmental Sustainability and Regulatory Challenges

**DOI:** 10.3390/nu17071245

**Published:** 2025-04-02

**Authors:** Lukas Nachtigall, Tilman Grune, Daniela Weber

**Affiliations:** 1 Department of Molecular Toxicology, German Institute of Human Nutrition Potsdam-Rehbruecke (DIfE), 14558 Nuthetal, Germany; 2 ProtinA (Joint BMEL Project DIfE/ATB), German Institute of Human Nutrition Potsdam-Rehbruecke (DIfE), 14558 Nuthetal, Germany; 3 ProtinA (Joint BMEL Project DIfE/ATB), Leibniz Institute for Agricultural Engineering and Bioeconomy (ATB), 14469 Potsdam, Germany; 4 Institute of Nutritional Science, University of Potsdam, 14469 Potsdam, Germany; 5 Food4Future (F4F), c/o Leibniz Institute of Vegetable and Ornamental Crops (IGZ), 14979 Grossbeeren, Germany

**Keywords:** edible insects, nutritional profile, protein quality, digestible amino acid scores, consumer acceptance, environmental sustainability, food safety

## Abstract

The scientific interest in edible insects as an alternative source of high-value protein for the human diet has increased drastically over the last decade. Edible insects harbour enormous potential in terms of planetary health. Their lower water and land use, lower feed conversion ratios, and overall lower global warming potential paired with a high nutritional value compared with conventional livestock are key drivers towards an environmentally sustainable diet. However, low consumer acceptance, as well as regulatory challenges, have slowed down the success of edible insects in Western countries, despite edible insects being consumed regularly all over the world. To date, four edible insect species have been approved as novel foods in the European Union—namely yellow mealworm (*Tenebrio molitor*), migratory locust (*Locusta migratoria*), house cricket (*Acheta domesticus*), and lesser mealworm (*Alphitobius diaperinus*). Depending on the species, they have a high protein content (48–67%), with a beneficial indispensable amino acid profile, high fat content (21–39%), with a high content of unsaturated fatty acids based on the dry matter, and contain reasonable amounts of minerals and vitamins. Unlike other animal-based foods, edible insects contain dietary fibre. Data on the bioavailability of nutrients in humans are scarce. Although numerous publications have investigated the nutritional profiles, environmental impacts, and future perspectives of edible insects, here, those findings are reviewed critically, as some publications were partially contradictory or related to selected species only. In this narrative review, we emphasise that edible insects could play a key role in a changing world with a steadily increasing demand for nutritionally valuable food and the depletion of natural resources.

## 1. Introduction

**Figure 1:**
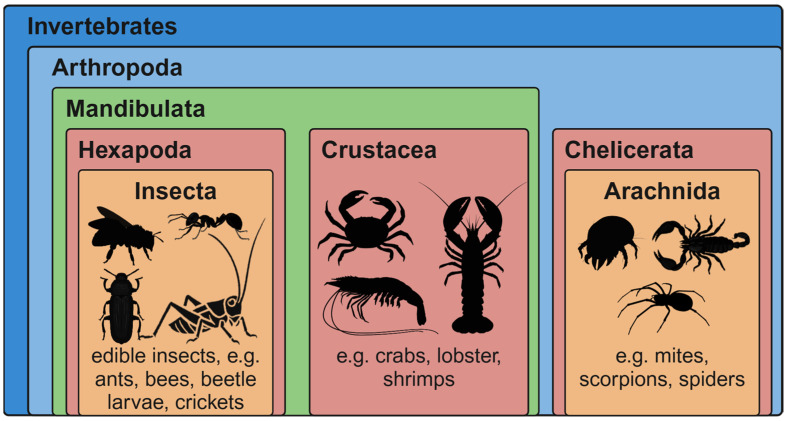
Simplified taxonomy of “insects”. In the animal group of invertebrates, the phylum Arthropoda contains, in addition to the subphylum Chelicerata with the class Arachnida, the clade Mandibulata. In this clade, there is the subphylum Myriapoda and the clade Pancrustacea, with the closely related subphyla Crustacea and Hexapoda. Insecta are a class of Hexapoda [2,3,4,12]. Created in BioRender. Nachtigall, L. (2025) https://BioRender.com/z02p871 (accessed on 27 March 2025).

Insects have always played a major role in the environment, economy, and science. More than one million species have been described, with a huge number still undiscovered [1]. The scientific understanding of Insecta differs from the colloquial use of the term “insects” as a collective term for all small invertebrates, which might include arachnids (Figure 1) [2]. However, the class of Insecta is situated independently in the subphylum Hexapoda, in the clade Mandibulata, and is only in the same phylum Arthropoda taxonomically [3,4,5]. The importance of insects for humankind could not be more fundamental. They pollinate flowering plants and degrade biological waste; silkworms produce silk, while drosophila melanogaster serves as key model system in science [6]—and insects are not a new phenomenon in human nutrition. Products, like honey and beeswax, from honeybees (*Apis mellifera*), carmine (E120—(EC) 1333/2008), a red dye from cochineals (*Dactylopius coccus*) and cooked whole insects, are a part of modern and traditional diets [1]. In fact, 1611 edible insect species were reported [7] after critically scrutinising previous estimations of 2111 species [8], with most of them being from tropical regions, where they tend to be larger and are available throughout the year [1]. In Africa, Asia, and Latin America edible insects were not only a part of traditional diets thousands of years ago, but are also consumed nowadays. It was estimated that 2 billion people from 113 countries around the world eat insects regularly—not as the sole source of nutrients, but rather as an addition to other food, whenever seasonally available [9,10]. However, this overestimation of people eating insects has been withdrawn recently. Newer estimations name figures of several hundred million people worldwide [11]. In Western countries, the consumption of insects is often perceived with disgust and as primitive [1].

The aim of this narrative review is to give a concise overview of the nutritional value of edible insects in general and those species that are approved as novel food in the European Union (EU) in particular, highlighting their high protein content and amino acid bioavailability. Beyond that, environmental sustainability and food safety aspects, as well as regulatory challenges within the EU related to edible insects, are discussed. The Google Scholar, PubMed, and Web of Science databases were searched between October 2024 and March 2025 using the search terms “*Acheta domesticus*”, “*Alphitobius diaperinus*”, “*amino acid*”, “*amino acid score*”, “*chemical composition*”, “consum*”, “*cricket*”, “edible insect*”, “*entomophagy*”, “environment*”, “*food*”, “*locusta migratoria*”, “*mealworm*”, “nutritional profile*”, “*protein*, *quality*”, and “*Tenebrio molitor*”, combined using Boolean connectors (AND, OR) to find remarkable, as well as very recent publications related to these issues. After a subjective review, additional information provided by the German Nutrition Society (DGE), the European Commission (EC), the Food and Agriculture Organization of the United Nations (FAO), and the World Health Organisation (WHO) was also included.

## 2. Regulations in the European Union

**Figure 2:**
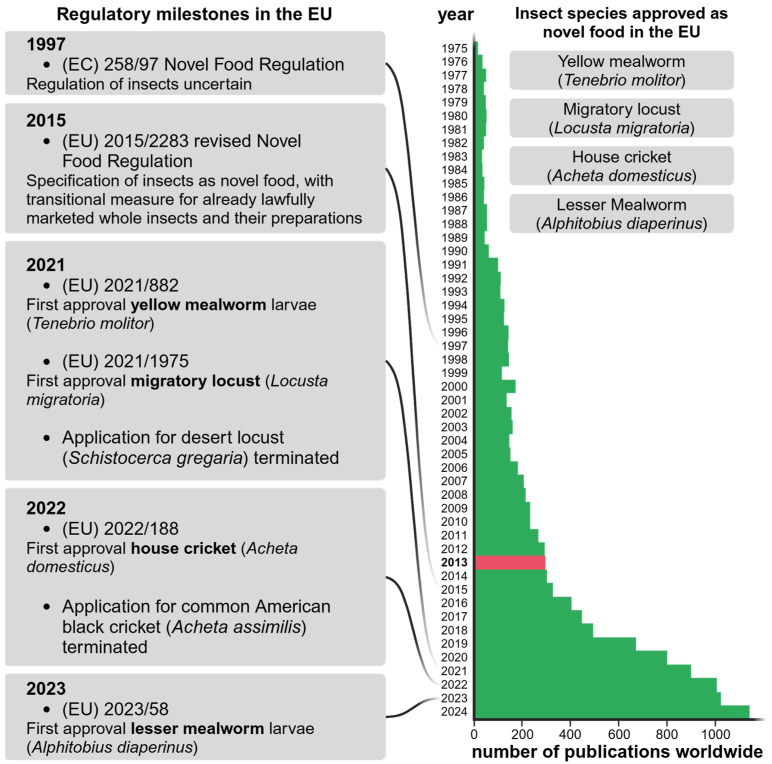
Number of publications worldwide, depending on their year of publication, based on a Web of Science search and regulatory milestones in the European Union (EU). Search terms were “*Acheta domesticus*”, “*Alphitobius diaperinus*”, “*amino acid*”, “*amino acid score*”, “*chemical composition*”, “consum*”, “*cricket*”, “edible insect*”, “*entomophagy*”, “environment*”, “*food*”, “*locusta migratoria*”, “*mealworm*”, “nutritional profile*”, “*protein, quality*”, and “*Tenebrio molitor*”, combined using Boolean connectors (AND, OR). Key publications were published in 2013 (red). Important regulatory milestones in the EU led to the approval of four insect species as novel food until 2025. Created in BioRender. Nachtigall, L. (2025) https://BioRender.com/6cjd03w (accessed on 27 March 2025).

Despite the perception of insects in Western countries, interest in edible insects as novel foods has increased drastically in the past 12 years (Figure 2). However, utilising the potentials of edible insects is not a new idea, as this was already proposed by Meyer-Rochow in 1975 [13]. Based on our Web of Science search from 24 March 2025, a total of 12,290 publications were published between 1932 and 2024, including studies on insect breeding and processing, the characterisation of different developmental stages, cultural and medical uses of insects, or studies on other insect species that were not included in this review. While several hundreds of articles have been published since the 1990s, publications by the FAO by van Huis et al. [1], van Huis [14], and Rumpold and Schlüter [15] that all were published in 2013 and have been cited thousands of times; these studies brought new impetus to edible insect research in Europe, with a huge impact. The number of publications increased from year to year, numbering over 1000 annually since 2022. In 2014, the EC requested a risk assessment on the safety of insects as food and feed from the European Food Safety Authority (EFSA). In the related scientific opinion, chemical, environmental, and microbiological hazards, including allergy potential and microbial or chemical contamination, were assessed [16].

In the EU, food that was not consumed in the EU before 15 May 1997 can only be introduced to the market after a safety assessment by the EFSA and approval by the EC under the first Novel Food Regulation ((EC) No 258/97). In 2015, this regulation was replaced by a revised Novel Food Regulation ((EU) 2015/2283), which became applicable on 1 January 2018. Until this new regulation, the legal status of insects as novel food had not been specified. This uncertainty led to the implementation of a transitional measure. As ruled by the European Court of Justice (C-526/19), whole insects were not regulated by the old novel food regulation. In that case, if an insect food product was lawfully introduced in an EU Member State, it could stay on the market, even if it would require a new authorisation under the new regulation until the EC made a decision in a new authorisation process requested until 2019 [10]. Examples of applications falling under this transitional measure from 2018 are the tropical house cricket (*Gryllodes sigillatus*), which was withdrawn in 2022 [17,18], honeybee drone brood (*Apis mellifera*), which was lawfully placed on the market in Finland, and whose EFSA safety assessment is still ongoing [19,20], and the lesser mealworm larvae (*Alphitobius diaperinus*), which was finally approved in 2023 [21,22,23].

The import of insects intended for human consumption into the EU is regulated in (EU) 2017/625 and amended by regulation (EU) 2019/625. In addition, some third countries (Canada, Switzerland, United Kingdom, South Korea, Thailand, and Vietnam) were authorised to export insects into the EU under regulation (EU) 2021/405, provided that these products were produced under EU standards of animal welfare, hygiene, and food safety [24].

Under this set of regulations many applications, of insect-based novel food products were submitted to the EC [23]. By January 2025, seven applications had been approved, resulting in the new commission implementing regulations and amendments to the European Union’s list of novel foods. Four insect species have been approved on the European market to date (Appendix A, Table A1) [25]. Dried yellow mealworm larva (*Tenebrio molitor*) was the first insect species ever to be approved as novel food within in the EU ((EU) 2021/882) on 1 June 2021 after a food safety assessment by the EFSA. Shortly afterwards, frozen and dried forms of the migratory locust (*Locusta migratoria*) were approved as second insect species ((EU) 2021/1975). In 2022, a second novel food application of yellow mealworm ((EU) 2022/169) was approved, followed by the introduction of frozen and dried forms as well as partially defatted powder of house crickets (*Acheta domesticus*) ((EU) 2022/188 and (EU) 2023/5). The last approved new insect species was lesser mealworm larva (*Alphitobius diaperinus*) ((EU) 2023/58) in 2023. In 2025, UV-treated powder of whole yellow mealworm was approved ((EU) 2025/89), as the EFSA had finished its safety assessment in 2023. Currently, risk assessments for the introduction of the black soldier fly larvae (*Hermetia illucens*) as the fifth approved novel food insect species are being carried out by the EFSA [26,27].

Prior to the end of 2024, two applications for hitherto unauthorised species, namely the desert locust (*Schistocerca gregaria*) and common American black cricket (*Acheta assimilis*), were terminated in 2021 and 2022, respectively [28].

## 3. About the Success of Insects as Novel Foods

Despite all regulatory efforts, the success of edible insects as novel food in Western countries has been a long time coming. Many “entopreneurs” and pioneer companies of insect foods [29] have emerged worldwide, but quite a few have already disappeared from the market again (compiled lists can be found in Ref. [30]) for various reasons.

### 3.1. Challenges of Insect Foods in Western Countries

The introduction of insect foods to Western markets is sometimes compared to the success of sushi after its establishment on the US market around the 1960s. Back then, the practice of eating raw fish together with other ingredients, like rice, soy sauce, and nori (seaweed) as sushi was part of Japanese cuisine, which was brought to the US by Japanese migrants. However, there are many reasons why “insects are not the new sushi” [31]. The insect-based foods approved in the EU are truly novel foods in these forms and do not have a history in cuisines in this form, neither in Western countries nor elsewhere. Additionally, in contrast to fish, which was generally eaten cooked even before the establishment of sushi, insects were not eaten, except for some special food items in recent history in Western countries [31,32]. However, the ancient Greeks and Romans described some insects being eaten as a delicacy. Even in religious literature, such as the Bible, insect consumption is mentioned [2]. Modern attempts to establish insect foods as meat replacements or snacks compete with already existing alternatives which additionally might be vegan, and altogether might not do justice to the potential of insects as food [31,32].

However, increasing consumer acceptance is an additional challenge that makes it unattractive for companies to tackle the legal hurdles in the first place. The refusal of consumers to eat insects could already be subconsciously influenced by the use of the Western term “entomophagy”. This somewhat unwieldy term labels this practice as abnormal and strange—notably, the consumption of crustaceans is not labelled as “crustaceaphagy” either [2,12]. Together with the concepts of insects as food contaminants, pathogens, or generally “unwanted”, a negative attitude has formed in Western cultures which leads to insect foods being met with disgust [1,12,33]. Disgust has been identified to be one of the major factors in the unwillingness to eat insects in surveys across different countries [34,35,36,37,38]. Other factors that influence the willingness to try insects are food neophobia, food safety, curiosity, and prior experience or tradition [12,39].

One strategy to overcome the disgust and food neophobia factors in Western countries could be to associate “edible insects” with positive connotations through catchy marketing slogans [40]. Another strategy could be incorporating insect-based ingredients into familiar foods, where the insects are no longer visible, such as pasta, protein shakes, or sweet and salty snacks [12,33], which was found to be more accepted than eating the whole insects [39]. For instances, in a German nationwide online survey, 41.9% of participants stated they would eat an insect burger, but only 15.9% would eat the whole insect the burger would be made out of [35]. But why should we consume a familiar product with insects as a new ingredient? Does this approach not encounter the same problem, of insects not having a clear place in Western cuisines, as described before?

One critical point that must also be considered is food safety, a parameter influenced by microbial aspects, toxico-chemical aspects, and allergenic potential. Due to the as yet non-standardised rearing and processing conditions of edible insects, the risk assessment is rather difficult [41]. In its risk profile on the safety of insects as food, the EFSA concluded that insect specific pathogens are not pathogenic to humans, partly due to the taxonomic distance between insects and humans. However, insects can act as transmitters for human pathogens via contaminated substrates or bad rearing conditions [1,16]. Thus, the EC classified farmed insects, destined for human consumption, as farmed animals, and thereby prohibits the use of side streams (ruminant proteins, food waste, or manure) as feed for the insects in regulation (EU) 2017/893, in order to reduce the biological hazards [42,43]. However, when mass rearing insects and due to the impossibility of removing the highly microbiologically colonised gut, the microbiological hazard can only further be reduced by good and hygienic rearing conditions and appropriate processing steps, such as thermal processing [44]. There is an ongoing debate concerning how conscious insects are and whether they have a sentience of pain. There is evidence that insects are aware of their surroundings and exhibit defensive behaviour when harmed. Regardless of how this question is answered, the rearing of insects as human food includes ethical aspects. Either way, precautionary adherence to animal welfare is advised to increase insect wellbeing and to harvest them as harmless as possible [41,45]. Rearing conditions, feed substrates, and the choice of insect species influence the toxico-chemical hazard. In addition to some insect species that produce their own toxins, the most critical sources for contamination are toxins, like pesticides or heavy metals from the feed substrates and, thus, need to be monitored routinely. As food processing could also influence the toxico-chemical hazard, insects may only be processed in accordance with the approved procedures in the EU (Appendix A Table A1) [16].

As shown in Figure 1, edible insects are closely related to crustaceans, some of which are also eaten around the world. Interestingly, the detailed phylogenetic relations between these groups are still being investigated [3,4,5]. In arthropods, also including house dust mites (relatives in the class Arachnida), several pan-allergens have been identified. These protein structures are highly conserved, resulting in cross-reactions between edible insects, crustaceans, and house dust mites. This is why people that have a food allergy to crustaceans or an inhalant allergy to house dust mites might also react allergic to edible insects. Among the most frequently mentioned pan-allergenic proteins are tropomyosin (muscle protein), arginine kinase and triosephosphate isomerase (enzymes), tubulin (cellular protein), and insect venom allergens [12,16,44,46]. The allergenicity of edible insect proteins was not observed to be removed after heat processing [12]. Although chitin itself is reported not to be allergenic, its immunomodulatory potential, both positive (antimicrobial and antitumor) and negative (enhancing allergic reaction), was described in the literature [16,44,47].

### 3.2. Edible Insects Around the World

Insects from various orders are eaten around the world, including (i) beetle larvae (Coleoptera), (ii) caterpillars (Lepidoptera), (iii) ants, bees, and wasps (Hymenoptera), (iv) crickets, grasshoppers, and locusts (Orthoptera). Traditionally, most of them are harvested from the wild [8,12].

(i) Beetle larvae: Palm weevil larvae (*Rhynchophorus* spp.) are harvested in tropic regions in Africa, southern Asia, and South America. Larvae from eggs, directly laid onto new palm tree leaves, can be collected and sold as a fried delicacy. Yellow mealworm (*Tenebrio molitor*) and lesser mealworm (*Alphitobius diaperinus*), approved as novel food in the EU, are not harvested from nature, but are instead reared under controlled conditions on an industrial scale [1,33].

(ii) Caterpillars: The fact that beetles are mainly not eaten in their adult stage also applies to butterflies and moths. For silkworms (*Bombyx mori*), in addition to the silk obtained, silkworm pupae are high in protein and, thus, are also eaten in Asia. In southern Africa, mopane caterpillars (*Gonimbrasia* spp.) are collected from mopane trees, dried, and eaten. They are high in protein, fat, and minerals. Similar practices can be found in Thailand and other Asian countries, where the bamboo caterpillar (*Omphisa fuscidentalis*) is consumed [1,33].

(iii) Ants, bees and wasps: Weaver ants (*Oecophylla* spp.), found in Africa, southern Asia, and Australia have been used as biocontrol agents in mango, citrus, or cacao plantations. In addition to their usage in pest management, their larvae and pupae are eaten. Honeybees (*Apis mellifera*) are semi-cultivated for the production of honey or beeswax, and the bee brood can also be eaten [1,33]. There is an ongoing EFSA risk assessment on honeybee drone brood as a novel food in the EU since the application was submitted in 2018 [20] (Appendix A Table A1).

(iv) Crickets, grasshoppers and locusts: In Latin America, chapulines (*Sphenarium* spp.), grasshoppers are caught and served as a street food. House cricket (*Acheta domesticus*) and migratory locust (*locusta migratoria*) are traditionally farmed at a small scale in Asian countries, especially Thailand. Since the approval of both species as novel food in the EU, they have also been reared in Europe in specialised farms [1,33].

### 3.3. Benefits of Eating Insects

The world population is expected to increase to ten billion people by the year 2050. Although, the population size in many central European and Asian countries is expected to have reached its peak and is projected to decrease, the population size in African and North American countries is projected to still increase [48,49]. At the same time, the food demand is estimated to increase by over 50%, while the demand for animal-based food in particular is expected to increase by 68% compared with 2010 [50]. This not only requires increased food production, but, even more importantly, it requires the production and equal distribution of nutritionally valuable food [48]. Furthermore, it demands changes in greenhouse gas (GHG) emissions, and in the use of land, water, and other limited resources in food systems, to tackle urgent environmental problems [39,51]. The current food system is a major factor of environmental degradation and demands a transformation of current diets. Currently 85% of soy products are used as animal feed, when they could instead also serve as an alternative protein source. Promising approaches include not only plant-based proteins from innovative farming concepts, as well as fungi, seaweed, micro- and macroalgae, or in vitro meat, but also edible insects. The integration of edible insects could represent a pathway towards a nutritionally balanced and environmentally sustainable diet [51,52,53].

**Figure 3:**
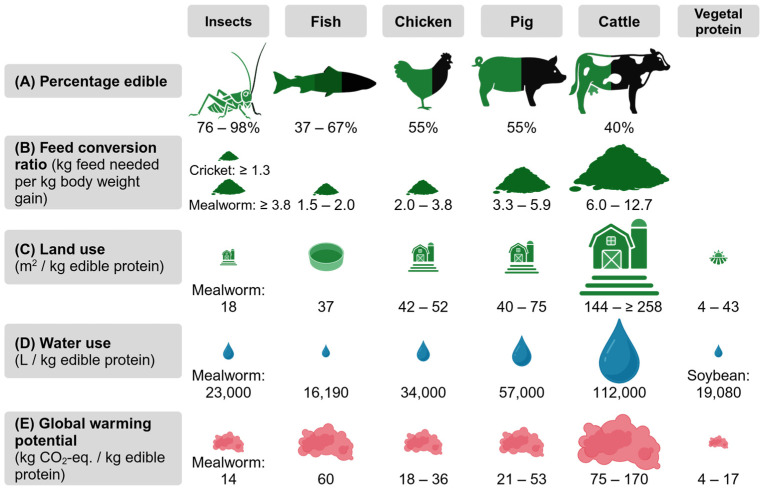
Benefits of edible insects as novel foods compared with conventional animal-based foods and vegetable protein (based on dry legumes and vegetable meat substitutes). (**A**) Percentage edible of the whole animal [1,52,54]. (**B**) Feed conversion ratio. Effectiveness depends on rearing conditions, substrate quality, age, and other factors [1,55,56]. (**C**) Land use (contains agricultural land needed for feed) [33,53,57,58,59]. (**D**) Water use (includes water needed for feed) [33,59,60,61]. (**E**) Global warming potential [33,53,57,58,59]. Created in BioRender. Nachtigall, L. (2025) https://BioRender.com/7fx26th (accessed on 27 March 2025).

In addition to these societal drivers, the consumption of edible insects is encouraged by their potential benefits for the environment (Figure 3).

It is estimated that 43% of all habitable land of the world is covered by agriculture and that 83% of all farmland is used for the production of animal-based food for humans. However, these animal-based foods only contribute 18% to the global calorie supply and 37% to the global protein supply, while the majority of protein comes from plant-based foods [59]. Seven percent of the global protein supply comes from the consumption of fish [62].

To produce 1 kg of edible insect-based protein, far less land is needed for rearing the insects compared with farmed fish, chicken, pigs, or cattle [53,57,58]. The production of insect-based foods also requires less water—another critical natural resource [60,61]. A significant amount of the water needed for animal-based food is attributable to the agriculture that is necessary to produce animal feed, i.e., feed that competes with human food [42].

As insects are cold-blooded, they do not need energy from the feed for the regulation of their body temperature, unlike livestock. However, to maintain habitable rearing conditions, heating the facilities might be necessary under certain circumstances. Low feed conversion ratios (amount of feed needed to gain a given weight) reflect this effective use of feed-based energy for insects. Depending on the feed value, to gain 1 kg of insect protein, far less feed is needed compared with chicken, pigs, or cattle, but the amount is comparable when it comes to fish. However, it should be noted that not only the crude protein content of a food determines its nutritional value, which can also differ depending on the processing grade. Up to 67% of fish, 55% of chicken and pigs, and only 40% of cattle is edible, while in contrast up to the whole insect (76–98%) is edible. Taken together, some insects could be twelve times more efficient than warm-blooded animals [1,52,54,55,56,63].

Furthermore, insects can also be reared on organic side streams as an attractive pathway fitting well into a circular economy concept. Insects could be fed with biomass (food “waste” or other organic by-products)—nutrients of low value that would normally be lost. Insects’ bioconversion into high value biomass would allow to recycle these losses back into the food supply chain. Thus, if food “waste” was used, it would not include the colloquial meaning of waste, i.e., being contaminated and dirty, but instead would need to be free of contaminants, such as heavy metals, mould, or pesticides. In the EU, however, insects for human consumption must be fed with eligible feed, which prevents the utilisation of such organic side streams due to safety concerns [42].

In regions where the ambient temperature is too low, the rearing facilities must be heated because insects are cold-blooded. Nevertheless, the energy use for rearing insects is still estimated to be lower than for other animal-based protein sources, but higher than for soybean [33]. Other publications argue that the energy use for rearing insects is comparable to farming chicken or pigs and is only lower compared with cattle [51,57].

Animal-based food contributes to 56–58% of all emissions caused by food [59]. Livestock alone contributes 10–18% to the total GHG emissions [1,64]. The production of insect foods emits far fewer GHGs, which is reflected by a lower global warming potential of up to ten times less compared with other animal-based protein sources and within the same range as soybean [33,53,57]. The benefits and challenges of insects as novel foods, as discussed above, are summarised in the following Table 1.

## 4. Nutritional Profile of Edible Insects

Edible insects have a high nutritional value for human nutrition. However, the nutritional compositions are highly variable between species—the same way as they are different between edible vertebrate species, plants, or fungi. Due to differing rearing conditions (feed composition, light, or temperature), developmental stage at harvest, or post-harvest processing steps, the measured content of macro- and micronutrients can even vary within the same species. As such, one might find very different nutritional profiles for a selected insect species to those presented in Table 2 and in the following section [12].

In general, edible insects have a high protein content with a satisfactory amino acid profile for human nutrition—comparable to conventional animal-based foods [47]. Based on dry matter weight, their protein content ranges between 35.3% (Isoptera—termites) and 61.3% (Orthoptera—crickets, locusts) [15]. In contrast, plant-based proteins often have an amino acid profile where one or more amino acids are missing in a sufficient quantity failing to meet the requirements of human nutrition. Altogether, intake of plant proteins has a lower anabolic potential than intake of animal proteins [52,53,65].

After protein, the second most important macronutrient of edible insects is fat. Based on their dry matter weight, their fat content ranges from 13.4% (Orthoptera—crickets, locusts), which is comparable to the fat content of dried soybeans, up to 33.4% (Coleoptera—beetles) [15,66]. However, the fatty acid composition differs to other fats of animal or vegetable origin, as discussed below. As such, most insect species substantially contribute to the total fat intake, which should account for around 30% of the total daily energy in healthy adults [67].

The least significant macronutrients come from carbohydrates, mainly chitin and glycogen. Nitrogen-free extract measurements revealed values of between 4.6% and 6.1% Odonata—dragonflies and Hemiptera—true bugs, respectively) and 22.8% (Isoptera—termites), which can be attributed to carbohydrates in a broader sense. The content of dietary fibre ranges from 5% (Hymenoptera—ants, bees, and wasps) up to 13.6% (Diptera—flies) and mainly comes from chitin [15]. This is where edible insects differ significantly from other edible animals, whose meat does not normally contain dietary fibre, and insects share similarities with vegetable protein sources, including fungi. Chitin is a modified nitrogen-containing polysaccharide, comparable to cellulose [68]. However, dried soybeans or fungi provide a higher portion of dietary fibre, i.e., around 22 g per 100 g [66]. Nevertheless, consuming edible insects could contribute to achieving the daily intake of at least 30 g of dietary fibre that is recommended by the DGE [67].

Additionally, edible insects are rich in minerals, such as iron, phosphorous, magnesium, copper, and zinc, as well as in some vitamins, most importantly vitamin B12 [12,47]. Some publications argue that edible insects also provide other bioactive compounds, such as antioxidants, enzyme inhibitors, antimicrobial peptides, and antinutrients [41,47,69].

**Table 2:** Macronutrient composition of edible insects approved in the EU compared with conventional livestock and vegetable protein sources.

Food	Energy (kcal/100 g)	Protein (g/100 g)	Fat (g/100 g)	Carbohydrates (g/100 g)	Fibre (g/100 g)	Reference
**Coleoptera (beetles) ^1)^**	490.30	40.69	33.40	13.20 ^2)^	10.74	[15]
*Tenebrio molitor* larvae **^1)^**	557.12	48.35	38.51	4.82 **^2)^**	8.48	[15]
*Alphitobius diaperinus* larvae **^1)^**	n.d.	48.60	29.60	n.d.	4.60	[70,71]
**Orthoptera (crickets, locusts) ^1)^**	426.25	61.32	13.41	12.98 ^2)^	9.55	[15]
*Acheta domesticus* **^1)^**	455.19	67.22	21.14	4.57 **^2)^**	19.18	[15]
*Locusta migratoria* **^1)^**	512.34	65.87	23.81	1.50 **^2)^**	12.78	[72,73]
Chicken, fresh meat, raw	165.87	19.90	9.60	0	0	[66]
Pork, fresh meat, raw	231.36	17.11	18.31	0	0	[66]
Beef, fresh meat, raw	155.59	19.60	8.58	0	0	[66]
Fish, fresh, raw	99.67	19.32	2.39	0	0	[66]
Soybean, dried	386.47	38.20	18.27	6.29	21.96	[66]
Wheat flour	348.47	10.04	0.98	72.34	2.75	[66]
Fungi, dried	291.11	48.24	2.92	6.58	22.32	[66]

^1)^ Values of edible insects are based on dry matter weight; ^2)^ Values are based on nitrogen-free extract (NFE = 100% − (protein + crude fat + ash + crude fibre + moisture)) [15]; n.d. not determined; Shown insect species belong to the respective order (bold) above.

### 4.1. Proteins and Amino Acid Profile

Some publications argue that the overall protein content of edible insects is generally overestimated by around 17%. This is due to the Kjeldahl method, which only measures the nitrogen content and, based on this, the protein content is calculated using a nitrogen-to-protein conversion factor. However, chitin also contains nitrogen, leading to an overestimation [74,75]. Nevertheless, the protein content can still be assumed to be high in edible insects with a beneficial amino acid composition for human nutrition (Table 3).

As proteins are the building blocks of life, the WHO has determined a daily requirement of 0.66 g protein per kg body weight for healthy adults, with adjustments for children, pregnant women, and elderly people [76]. The DGE even recommends a daily protein intake of 0.8 g/kg body weight for healthy adults and 1.0 g/kg body weight for elderly people [67]. Proteins are the structural and functional units of the human body and are abundant in every cell. Thus, the daily protein supply from the diet is required for the synthesis of endogenous proteins to maintain whole-body metabolic health [53,77]. However, it is not just the quantity of protein that is important, but also the quality of the protein, i.e., the amino acid profile, as shown in Table 3.

Some of the 21 proteinogenic amino acids cannot be synthesised by the human body and, thus, must be taken up with the diet. The lack of these indispensable amino acids is a form of malnutrition that can lead to stunted physical growth in infants and children, fatigue, increased vulnerability to infectious diseases, and an increased mortality over time. Dispensable amino acids can be synthesised endogenously, but some of them need indispensable amino acids as precursor molecules for that, which makes them conditionally indispensable. For instance, cysteine can be synthesised from methionine and tyrosine can be synthesised from phenylalanine, but not vice versa. Thus, the intake of dispensable amino acids is no less important [77,78].

In general, the protein content in animal-based foods is higher than in plant-based foods. Cereals have a protein content between 7–13%. Fresh meat from conventional livestock or fish contain nearly double the amount of protein and are even surpassed by soy (around 38%) [77]. As shown in Table 3, the content of the indispensable amino acids of livestock and fish exceeds the daily requirements recommended by the WHO, and these foods are rich in lysine in particular [66,76]. Unlike other plant-based proteins, protein from soybeans has an amino acid profile comparable with animal proteins; however, it is low in methionine. In contrast, wheat and other cereal proteins are deficient in lysine, threonine or tryptophan in terms of WHO recommendations [65,66,76]. Fungi are low in methionine and phenylalanine. To cover the requirements anyway, complementary sources of indispensable amino acids need to be mixed in the diet. For example, cereals (low lysine) and legumes (low methionine) complement each other [77].

In general, the protein content and amino acid profile of insect-based protein is comparable to that of other animal-based protein sources and meets the daily requirements of indispensable amino acids [15,79]. It was reported that the total protein content increases with the development progression of an insect [80]. That was reflected in lower protein content in the beetle larvae than in adult crickets or locusts, as shown in Table 2. While the content of indispensable amino acids of *Tenebrio molitor* larvae, *Alphitobius diaperinus* larvae, and *Acheta domesticus* generally meets or exceeds the recommended minimum intake, it is only sufficient or lower for *Locusta migratoria*. The amino acid profile, however, also depends on the feeding substrate, which is primarily plant-based [12,15,76]. Nevertheless, the amino acid profiles of the species shown in Table 3 from the same order are very similar to each other. Depending on the species, some edible insects are low in cysteine, methionine, or tryptophan, but rich in lysine or threonine [15,79]. However, some researchers also argue that these measured low contents of the mentioned indispensable amino acids are also attributable to the analysis method [80]. The digestibility and bioavailability of proteins and amino acids from edible insects is discussed in the next section.

### 4.2. Lipids

The fatty acid composition of edible insects is dependent on the feed to some degree, just like the amino acid composition. In general, edible insects contain more unsaturated fatty acids than saturated fatty acids and their profile is comparable to those of fish and poultry [15,81]. As in other animal-based foods, the most abundant saturated fatty acids are palmitic acid (C16) and stearic acid (C18), but the polyunsaturated fatty acids linoleic acid (C18:2 *n*-6) and α-linolenic acid (C18:3 *n*-3) are also found in edible insects [15,69]. In contrast, soy and other plant-based fat sources, are low in saturated fatty acids, are but rich in mono- and polyunsaturated fatty acids [65]. As shown in Table 2, the total fat content of the beetle larvae is significantly higher than in the adult crickets or locusts. The phenomenon that the total fat content decreases with progressing development was also found to be true for insects in general [80].

Insects cannot synthesize cholesterol de novo but instead take up the sterols needed for the conversion to cholesterol for their cell membranes or hormones with the feed. Thus, edible insects can contain cholesterol, as do other animal-based foods. In contrast, vegetable protein sources, such as soy or fungi, are low in or do not contain dietary cholesterol [65,66,69]. It was reported that not all insect species contain cholesterol and that the cholesterol content could be influenced by the feed [15].

### 4.3. Micronutrients and Bioactive Compounds

The mineral and vitamin content of edible insects depends on their developmental stage and feed; therefore, highly variable information can be found in literature [69]. However, edible insects are reported to have a low content of calcium but are reported to be rich in iron, phosphorous, magnesium, copper, and zinc [12]. Insects can contain vitamins, such as vitamin A, B1 (thiamine), B2 (riboflavin), B3 (niacin), B5 (pantothenic acid), B7 (biotin), B12 (cobalamin), and C [12,15,47]. However, they are not proving to be an effective source unless the insects are specially treated. As approved by the EC in 2025 (Appendix A Table A1), for the first time, an insect-based product, specifically yellow mealworm larvae powder, can be radiated with UV-B light, thereby enhancing the vitamin D3 (cholecalciferol) content [82].

Additionally, edible insects can contain bioactive compounds that could have an impact on human health. Chitin and its deacetylated derivate chitosan, for example, are said to have antimicrobial, antioxidant, and prebiotic effects [69]. On the other hand, chitin, as a source of dietary fibre, could function as an antinutritional factor that affects the bioavailability of minerals. As described before, chitin could also act as an immunomodulator, and, in the worst cases, could aggravate an allergic reaction [16,47,69]. Due to the high protein content in edible insects, antioxidative properties have been described in in vitro studies [41]. Furthermore, it was reported that insect hydrolysates could have an Angiotensin-I converting enzyme (ACE) inhibitory activity. Angiotensin II, which is produced by the ACE, has a vasoconstrictive effect. If this really is inhibited by insect protein, insect-based foods could prevent the elevation of the blood pressure, potentially preventing cardiovascular diseases [41,69]. Some researchers even argue that the consumption of edible insects could ameliorate obesity, type 2 diabetes mellitus, and even metabolic syndrome [68]. Chitin was already mentioned as a potential antinutrient. Alkaloids, oxalate, phytate, and tannin represent others that are found in edible insects [47,69]. However, in these fields, further research is necessary to validate potential effects in vivo.

## 5. Bioavailability

The nutritional profile (corresponding to the biological value) of edible insects has been studied extensively, but with less attention paid to bioavailability. Bioavailability is the term used to describe the proportion of nutrients of a food that is actually available to the body after digestion and intestinal absorption [47]. One prominent example of how the kind of food can influence the bioavailability is legumes. Some legumes contain a trypsin inhibitor which can reduce the ileal digestibility of proteins when consumed. As mentioned above, other antinutritive factors can reduce the bioavailability of micronutrients [65]. The same principle applies to edible insects and other foods.

The biological value of edible insects is often determined on the basis of their dry matter mass and often favours edible insects in comparison to other foods. However, the dry matter does not necessarily represent the form in which insects are actually consumed. The dry matter mass can vary between 32.4% (cricket) and 71.9% (termite) in relation to the fresh weight [83].

Measuring bioavailability directly in humans is difficult and expensive [84]. Thus, animal studies on growth performance and nutrient status after feeding with insect products were performed and reviewed systematically by others. The inclusion of insects into the feed improved the growth performance in broiler chickens and rabbits. However, in malnourished rats, the supplementation of insects into the feed to recover the weight and nutritional status resulted in results similar to those in the control group. As these results showed at least the same effect as other dietary protein sources, this approach of exchanging conventionally used protein sources with insect protein could find application in complementary foods for infants and children. Studies in mice and hens showed a reduction in serum triglyceride and cholesterol levels after feeding the animals with insects; however, the reducing effects on blood pressure were inconsistent [85].

The FAO and WHO have developed two scores to assess the dietary protein quality for human nutrition. The first was the PDCAAS (protein digestibility-corrected amino acid score), which was replaced in 2011 by the DIAAS (digestible indispensable amino acid score) [86,87].

In brief, the PDCAAS is based on the true faecal digestibility of indispensable amino acids of the whole test protein in relation to the reference amino acid pattern (Table 3 [76]). The true faecal digestibility is determined by relating the nitrogen content in the faeces after consumption of the test protein to the original nitrogen content in the test protein. As has been already explained, the protein content can be determined using a nitrogen-to-protein conversion factor. The PDCAAS is then calculated using the following Equation (1), using the values of the true faecal digestibility in humans for different foods, which are available from the literature [76,87]:

(1) $$\mathrm{PDCAAS}=\frac{\mathrm{mg}\;\mathrm{of}\;\mathrm{amino}\;\mathrm{acid}\;\mathrm{in}\;1\;g\;\mathrm{of}\;\mathrm{test}\;\mathrm{protein}}{\mathrm{mg}\;\mathrm{of}\;\mathrm{same}\;\mathrm{amino}\;\mathrm{acid}\;\mathrm{in}\;\mathrm{requirement}\;\mathrm{pattern}}\;\cdot \;\mathrm{true}\;\mathrm{faecal}\;\mathrm{digestibility}$$

The indispensable amino acid with the lowest PDCAAS is considered as the first limiting amino acid of the test protein. This means that the indispensable amino acid with the lowest PDCAAS is not present in the food in sufficient amounts to meet the requirement for adequate intake. Beyond that, the requirement of the second, third, and so forth limiting amino acid might not be met either. Thus, more of that food (or other food that contains more of the respective amino acid) should be consumed accordingly to ensure a sufficient supply. However, when the required intake of the first limiting amino acid is met, then the requirement of the other indispensable amino acids is met as well. PDCAAS values above 1 are truncated, as it was believed that the intake of amino acids at more than the required level would not have an additional physiological value. However, with that approach, the nutritional value of high-quality protein sources is underestimated. Additionally, antinutritive factors in certain foods, which limit not only the absorption of micronutrients but also of proteins, are not considered, leading to the endogenous loss of amino acids, which would be reflected in a higher PDCAAS. As discussed before, the nitrogen-to-protein-conversion factor might differ between certain types of foods [84,88].

To overcome these methodical issues and to incorporate the fact that the digestibility of individual amino acids can differ, the PDCAAS was replaced by the DIAAS.

In brief, the DIAAS is based on assessing the intestinal absorption (true ileal digestibility) of indispensable amino acids in relation to the reference amino acid pattern (Table 3 [76]). To determine the true ileal digestibility of a certain food, the amino acid profile in the ileal digesta must be measured after consumption of the test protein. It is recommended to determine the true ileal digestibility values in growing pigs or in laboratory rats when these rates are not available from humans. The DIAAS is then calculated using the following Equation (2) [86,88]:

(2) $$\mathrm{DIAAS}=\frac{\mathrm{mg}\;\mathrm{of}\;\mathrm{amino}\;\mathrm{acid}\;\mathrm{in}\;1\;g\;\mathrm{of}\;\mathrm{test}\;\mathrm{protein}}{\mathrm{mg}\;\mathrm{of}\;\mathrm{same}\;\mathrm{amino}\;\mathrm{acid}\;\mathrm{in}\;\mathrm{requirement}\;\mathrm{pattern}}\;\cdot \;\mathrm{true}\;\mathrm{ileal}\;\mathrm{digestibility}$$

As for the PDCAAS, the amino acid with the lowest DIAAS is considered as the first limiting amino acid of the test protein. In contrast to the PDCAAS, there is no truncation [86].

However, true ileal digestibility values are not widely available for novel foods, including edible insects. Thus, the PDCAAS is often still used over the DIAAS. An alternative way to assess the true ileal digestibility other than in humans or animals is to use a harmonized static in vitro digestion model (INFOGEST [89]) that simulates the salivary, gastric, and intestinal phase of human digestion by utilising multiple digestive enzymes that are necessary in the respective phase. Based on this, an in vitro DIAAS can be determined [90]. The PDCAAS and DIAAS values of edible insects approved in the EU and other common animal- and vegetable-based foods are compared in the following Table 4.

The values of PDCAASs and DIAASs from the literature are highly variable and inconsistent between methods. Additionally, many publications calculated the scores for certain age-groups using different amino acid reference patterns provided by the FAO/WHO [76,86,87] only. The PDCAASs for adults of edible insects were comparable to each other, ranging between 0.76, 0.84, and 0.86 for *Alphitobius diaperinus* larvae, *Acheta domesticus*, and *Tenebrio molitor* larvae, respectively. Only the PDCAAS of *Locusta migratoria* was significantly lower, with a value of 0.44. However, the amino acid reference pattern of preschool children was used in this case [91,94,95]. The DIAAS of *Tenebrio molitor* larvae was 64.0 for in vivo data and higher (94.4 and 106.4) for in vitro data [88,92,93]. For the other edible insects, the DIAAS values were 83.0 in *Alphitobius diaperinus* larvae, 86.2 in *Locusta migratoria*, and 89.0 (in vivo)—88.5 and 119.3 (in vitro) for *Acheta domesticus* [88,92,93]. For the most of these insect species, the DIAAS was comparable and in the same range as the PDCAAS, with methionine and cysteine being the first limiting amino acid when the amino acid reference pattern for adults was used. This was also reflected in their amino acid profiles shown in Table 3. Compared with other animal-based foods, the PDCAAS as well as the DIAAS values of edible insects were lower. However, the scores were significantly higher than in plant-based proteins. As shown in the amino acid profiles (Table 3), wheat and soy are low in lysine and methionine, which was reflected in them being the first limiting amino acids. Soy protein isolate and whey protein concentrate have high scores around 1 and above and, therefore, have an exceptionally high nutritional protein quality [98].

The incomplete digestibility might also be a reason of the allergenic potential of edible insects, as the allergens are not broken down to low-molecular levels (small peptides or amino acids). Instead, allergens can escape the digestive system and provoke sensitisation [99]. The allergenicity of foods and the bioavailability of nutrients not only depend on the nutritional profile but are also influenced by processing. It is generally assumed, that the removal of chitin and protein isolation improves the digestibility [84,100]. However, it was also observed that the in vitro DIAAS was reduced when chitin-reduced *Acheta domesticus* or *Tenebrio molitor* were used [93]. Several studies have investigated the influence of household- and industrially used cooking techniques. However, the results were contradictory. Some studies reported a decreased protein digestibility, while others did not see an effect and some found significantly increased digestibility compared with non-processed edible insects [100].

Only a few human studies have investigated the effect of the consumption of edible insects on the amino acid levels in the blood. Based on them, it can be assumed that edible insects are a slowly digestible protein source compared to whey as a control. Furthermore, the changes in the blood amino acid levels over time were comparable to these of vegetable protein sources, such as soy and pea [101,102,103,104].

A complex and expensive method to assess the protein quality directly in humans would be to create intrinsically isotope-labelled test foods that are consumed to then determine the true ileal amino acid digestibility [88].

To summarise, some studies have already conducted in vitro and in vivo investigations on edible insects. The protein bioavailability of the insects that are approved as novel foods in the EU is higher than from plant-based protein and might be slightly lower than from other common animal-based protein. However, the methods used to determine the bioavailability varied, making the results less comparable. Additionally, there is a lack of data from human studies which should be the focus of future research in order to initiate changes in food systems.

## 6. Conclusions and Future Prospects

The world faces a double burden in terms of nutrition: on the one hand, in 2023, the worldwide number of undernourished people rose to 733 million; on the other hand, in 2022, 881 million adults worldwide faced obesity, and the trend is rising [105]. Additionally, food systems must change, to provide more nutritionally valuable food, to face the double burden and to become more environmentally sustainable [48,51]. Could the consumption of edible insects be the key to resolving this issue?

While the interest in edible insects in wealthy, Western regions was triggered by economic and environmental reasons, the regions that could benefit most from this are those with food insecurity, malnutrition and poverty.

Two systematic reviews came to the conclusion that human intervention studies are scarce [68,85]. Some studies focussed on insects as an ingredient in new complementary foods for infants and children, based on previous insights from animal studies. While they found that the addition of edible insects can assumed to be safe, this incorporation was predominantly associated with neutral results, as it was concluded that complementary foods containing insects could promote growth in the same way as conventional complementary foods. However, incorporating locally available edible insects could be a huge benefit where other expensive animal-based food sources are inaccessible, especially as a source of vitamin B12, which is more or less exclusively of animal origin. Additionally, this addition could be beneficial to prevent iron-deficiency anaemia [68,69].

In general, it was observed that insect consumption had prebiotic effects and provided amino acids comparable to soy [68,85]. As shown in Figure 3, the production and consumption of edible insects could have a 30–90% lower environmental impact in terms of land use, water use, and global warming potential compared with conventional livestock. Furthermore, they are a good source of animal-based protein, with a beneficial amino acid composition, as well as fat, minerals, and vitamins (Table 2 and Table 3). In Western countries, however, only around 25% of people are willing to try edible insects, with the biggest concern being disgust [12]. Consumer acceptance, accessibility, and affordability must be improved in order to establish edible insects in Western diets in interests of environmental sustainability [85].

As attempts to establish insect-based foods turned out to be difficult, new strategies are required. Perhaps the aim should not be to replace already existing alternative food sources, but instead to use edible insects as a functional ingredient, able to enhance the nutritional profile of a food—just like the addition to complementary foods [31,32]. Some companies have already made great efforts in this respect around the world (see [30]), and in particular in the EU, to comply with the strict regulations that are in place (Appendix A Table A1). The fields of application cover baked goods (biscuits), vegetable-based dishes (canned legumes, salads), imitation meats, pasta, pizza, sausages, sports products (protein powder), sweet and salty snacks (chips, chocolate), or simply whole insects [32].

In this narrative review we conclude that, while insects provide a variety of promising opportunities, more research is necessary to validate previous findings and overcome legitimate concerns. The nutritional profiles of the edible insects approved in the EU are comparable to those of conventional livestock, especially in terms of protein quality. However, they promise significant benefits in many aspects of environmental sustainability. As the bioavailability of indispensable amino acids from edible insects is inferior to the bioavailability from livestock, innovative post-harvest processing techniques are required to enhance the nutritional value of those insects. The effects of these new processing techniques on the bioavailability of amino acids should be investigated in vitro and validated in humans in vivo.

## Figures and Tables

**Table 1:** Benefits and challenges of insects as novel foods compared with conventional animal-based foods in Western cultures.

Benefits	Challenges
Worldwide traditions of eating wild edible insects	(International) legislation
Less land use (with some species requiring 14 times (or more) less land than livestock [33,57,58])	Edible insect-based novel foods have no clear place in Western cuisine
Lower water use (five times (or more) less than livestock depending on insect species [33,60,61])	Consumer acceptance (disgust, food neophobia, curiosity, tradition)
Lower feed conversion ratios (up to twelve times less feed is needed to gain 1 kg of edible insects compared with livestock [1,55])	Food safety (standardized rearing conditions, contamination, insect welfare)
Bioconversion of organic side streams to high-value food (circular economy)	Allergenicity
Lower global warming potential (ten times lower than livestock depending on the insect species [33,57,58])	
High nutritional value for human nutrition	

**Table 3:** Amino acid profiles in mg per g of crude protein of edible insects approved in the EU compared with conventional livestock, vegetable protein sources, and the daily requirement of indispensable amino acids in human nutrition.

**Food**	**Indispensable Amino Acids (mg/g Protein)**									**Reference**
	**His**	**Ile**	**Leu**	**Lys**	**Met**	**Phe**	**Thr**	**Trp**	**Val**	
**Coleoptera (beetles) ^1)^**	26.3	45.6	74.2	50.6	16.2	47.1	35.2	10.1	51.9	[15]
*Tenebrio molitor* larvae **^1)^**	34.9	48.8	88.8	60.1	15.5	39.9	39.1	9.2	64.7	[15]
*Alphitobius diaperinus* larvae **^1)^**	39.7	46.1	73.2	70.5	15.9	51.7	43.1	14.7	57.6	[70]
**Orthoptera (crickets, locusts) ^1)^**	21.2	39.6	74.8	53.9	19.3	46.6	35.8	8.1	50.3	[15]
*Acheta domesticus* **^1)^**	23.1	41.2	83.4	52.4	17.3	31.0	35.6	7.0	50.3	[15]
*Locusta migratoria* **^1)^**	14.0	25.0	46.0	29.0	16.0	37.0	19.0	4.0	41.0	[73,74]
Chicken, fresh meat, raw	26.6	56.2	77.5	88.8	27.9	39.6	44.0	12.2	51.4	[66]
Pork, fresh meat, raw	38.7	49.5	75.5	86.3	27.9	38.2	49.0	11.7	55.9	[66]
Beef, fresh meat, raw	33.0	49.6	80.8	83.8	24.8	41.0	44.9	11.1	54.3	[66]
Fish, raw	22.9	49.5	84.6	99.5	34.6	42.0	46.3	11.7	53.2	[66]
Soybean, dried	21.7	46.6	74.3	49.7	15.2	51.6	39.0	11.8	46.1	[66]
Wheat flour	19.2	40.2	71.8	21.0	14.8	48.2	28.0	10.6	42.9	[66]
Fungi, dried	20.4	39.4	43.1	60.8	8.3	26.5	31.2	8.5	32.1	[66]
Daily amino acid requirement in human nutrition	15	30	59	45	16	38 ^2)^	23	6	39	[76]
										
	**Dispensable Amino Acids (mg/g Protein)**									
	**Ala**	**Arg**	**Asx**	**Cys**	**Glx**	**Gly**	**Pro**	**Ser**	**Tyr**	
**Coleoptera (beetles)** ^1)^	69.5	53.9	n.d.	14.6	123.7	55.2	64.1	42.6	55.7	[15]
*Tenebrio molitor* larvae ^1)^	79.1	56.1	81.0	9.2	123.2	56.4	69.8	51.8	77.4	[15,74]
*Alphitobius diaperinus* larvae ^1)^	65.8	53.5	93.8	9.6	130.1	42.0	63.6	44.1	84.9	[70]
**Orthoptera (crickets, locusts)** ^1)^	77.4	53.6	n.d.	12.8	64.5	54.0	53.9	41.9	61.5	[15]
*Acheta domesticus* **^1)^**	82.4	59.2	93.0	9.1	104.7	48.0	55.2	50.9	46.4	[15,74]
*Locusta migratoria* **^1)^**	75.0	37.0	43.0	8.0	68.0	40.0	76.0	23.0	58.0	[73,74]
Chicken, fresh meat, raw	62.7	60.6	98.9	13.1	160.7	61.0	45.7	40.1	33.1	[66]
Pork, fresh meat, raw	60.3	59.8	95.1	11.8	153.0	55.9	47.0	43.7	40.6	[66]
Beef, fresh meat, raw	66.2	59.9	93.3	11.5	162.8	60.4	47.8	41.8	33.0	[66]
Fish, raw	60.1	62.8	106.4	12.8	157.6	44.7	33.0	43.1	35.1	[66]
Soybean, dried	40.1	61.8	104.5	15.4	169.9	37.2	47.6	44.2	32.7	[66]
Wheat flour	32.4	37.6	42.0	21.0	320.5	36.8	127.0	57.8	28.0	[66]
Fungi, dried	112.9	71.6	104.1	5.1	158.4	61.3	124.8	67.7	23.6	[66]

^1)^ Values of edible insects are based on dry matter weight; **^2)^** Sum of Phe and Tyr; n.d. not determined; Shown insect species belong to the respective order (bold) above; Ala, alanine; Arg, arginine; Asx, asparagine and aspartic acid; Cys, cysteine; Glx, glutamine and glutamic acid; Gly, glycine; His, histidine; Ile, isoleucine; Leu, leucine; Lys, lysine; Met, methionine; Phe, phenylalanine; Pro, proline; Ser, serine; Thr, threonine; Trp, tryptophan; Tyr, tyrosine; Val, valine.

**Table 4:** PDCAAS (protein digestibility-corrected amino acid score) and DIAAS (digestible indispensable amino acid score) for adults and the first limiting amino acid of edible insects approved in the EU compared with common animal- and vegetable-based foods.

Food	PDCAAS	DIAAS	First Limiting Amino Acid	Reference
*Tenebrio molitor* larvae	0.86 ^1) c)^		Met + Cys	[91]
		64.0 ^2)^	Met + Cys	[88]
		94.4 ^3)^	Lys	[92]
		106.4 ^3)^	Met + Cys	[93]
*Alphitobius diaperinus* larvae	0.76 ^1)^		n.d.	[94]
		83.0 ^2)^	Met + Cys	[88]
*Acheta domesticus*	0.84 ^1) c)^		Leu	[91]
		89.0 ^2)^	Met + Cys	[88]
		88.5 ^3)^	Met + Cys	[92]
		119.3 ^3)^	Leu	[93]
*Locusta migratoria*	0.44 ^1) ps)^		Trp	[95]
		86.2 ^3)^	Met + Cys	[92]
Chicken breast	1.01 ^2)^	108.0 ^4)^	n.d.	[96]
		136.5 ^3)^	Leu	[93]
Pork		119.0 ^2)^	none	[97]
Beef	0.92 ^4)^		n.d.	[87]
		119.0 ^2)^	none	[97]
Soy protein isolate	1.02 ^2)^	98.0 ^2)^	Met + Cys	[98]
Tofu	0.56 ^2)^	52.0 ^3)^	Met + Cys	[53]
Wheat	0.51 ^2)^	54.0 ^2)^	Lys	[98]
Whey protein concentrate	1.34 ^2)^	133.0 ^2)^	His	[98]

^1)^ investigated in rats; ^2)^ investigated in growing pigs; ^3)^ investigated in vitro; ^4)^ species in which the digestibility coefficients which were investigated are unknown; ^c)^ an amino acid reference pattern for 6-month to 3-year-old children was used [86]; ^ps)^ amino acid reference pattern for preschool children was used [87]; n.d. not determined; Cys, cysteine; His, histidine; Leu, leucine; Lys, lysine; Met, methionine; Trp, tryptophan.

## Appendix A

**Table A1:** Overview of the status of all applications for insect-based products as novel foods within the European Union (EU) as of January 2025 (listed by the European Commission [23]). Link between EU application number, European Food Safety Authority (EFSA) question number, EFSA safety assessment output (if available), and commission implementing regulation [25]. Highlighted applications have received approval to be placed on the market.

Year of Application	Status of Application	Product and Species	EU Application Number	EFSA Question Number and Output
2018	Approval as a novel food in 2023 (EU) 2023/58	Frozen and freeze-dried forms of lesser mealworm larvae (*Alphitobius diaperinus*)	NF 2018/0125 [21]	EFSA-Q-2018-00282 [22,106]
2018	Ongoing/positive EFSA safety assessment available	Frozen, dried, and powder forms of house crickets (*Acheta domesticus*)	NF 2018/0128 [107]	EFSA-Q-2018-00543 [108,109]
2018	Approval as a novel food in 2021 (EU) 2021/882	Dried yellow mealworms (*Tenebrio molitor*)	NF 2018/0241 [110]	EFSA-Q-2018-00262 [111,112]
2018	Application withdrawn in 2022	Dried tropical house crickets (Gryllodes sigillatus)	NF 2018/0260 [17]	EFSA-Q-2018-00263 [18]
2018	Ongoing EFSA risk assessment	Heat-treated locust nymphs or adults of migratory locusts (*Locusta migratoria*)	NF 2018/0395 [113]	EFSA-Q-2018-00513 [114]
2018	Ongoing/positive EFSA safety assessment available	Frozen and dried forms of whole yellow mealworm larvae (*Tenebrio molitor*)	NF 2018/0396 [115]	EFSA-Q-2018-00746 [116,117]
2018	Ongoing EFSA risk assessment	Honeybee drone brood (*Apis mellifera*)	NF 2018/0754 [19]	EFSA-Q-2019-0020 [20]
2018	Application withdrawn in 2024	Black soldier fly (*Hermetia illucens*) meal	NF 2018/0765 [118]	EFSA-Q-2019-00046 [119]
2018	Approval as a novel food in 2022 (EU) 2022/169	Frozen, dried, and powder forms of yellow mealworm larvae (*Tenebrio molitor*)	NF 2018/0802 [120]	EFSA-Q-2019-00101 [121,122]
2018	Approval as a novel food in 2021 (EU) 2021/1975	Whole and ground migratory locusts (*Locusta migratoria*)	NF 2018/0803 [123]	EFSA-Q-2019-00115 [124,125]
2018	Approval as a novel food in 2022 (EU) 2022/188	Whole and ground house crickets (*Acheta domesticus*)	NF 2018/0804 [126]	EFSA-Q-2019-00121 [127,128]
2019	Approval as a novel food in 2023 (EU) 2023/5	Defatted whole house cricket powder (*Acheta domesticus*)	NF 2019/1227 [129]	EFSA-Q-2019-00589 [130,131]
2019	Approval as a novel food in 2025 (EU) 2025/89	UV-treated powder of whole yellow mealworm larvae (*Tenebrio molitor*)	NF 2019/1142 [132]	EFSA-Q-2019-00748 [82,133]
2020	Ongoing/positive EFSA safety assessment available	House cricket powder (*Acheta domesticus*)	NF 2020/1860 [134]	EFSA-Q-2021-00262 [135,136]
2020	Ongoing EFSA risk assessment	Protein-rich flour from yellow mealworm larvae (*Tenebrio molitor*)	NF 2020/1959 [137]	EFSA-Q-2021-00105 [138]
2021	Ongoing EFSA risk assessment	Vitamin D3-containing UV-treated mealworm oil	NF 2021/0039 [139]	EFSA-Q-2022-00534 [140]
2023	Ongoing EFSA risk assessment	Dried defatted powder of *Hermetia Illucens* larvae	NF 2023/15216 [141]	EFSA-Q-2023-00703 [26]
